# Privacy online: up, close and personal

**DOI:** 10.1007/s12553-017-0197-z

**Published:** 2017-07-07

**Authors:** Eneken Tikk

**Affiliations:** 59 van Lansbergestraat, 2593 SB Den Haag, Netherlands

**Keywords:** Data protection, Privacy, Security, Responsibility

## Abstract

In the era of information, administration of personal data protection mingles with expectations of access to information as well as the overall sense of cyber (in)security. A failure to appropriately consider the *system* of data processing relationships easily reduces personal data protection to assurances in letter. The complexity of contemporary data transactions demands a systemic and structured normative approach to personal data protection. Any evaluation of relevant norms should not be isolated from factors that determine or condition their implementation. As privacy is an intrinsically subjective claim, enforcing data privacy is premised on data subject’s personal participation in the protection of her data.

## Introduction

Privacy is a much-addressed *problematique*, intensified against the backdrop of development and propagation of information technologies, their convergence with telecommunication and electronics as well as socio-technical trends like big data, cloud, and the Internet of Things (IoT). Normative^1^ guarantees to personal data^2^ protection need to stand the test of their time to guarantee informational self-determination in the context of online data processing^3^ and automated decision-making.^4^

In the era of commodification of all data [2], administration of personal data protection is intrinsically linked to societal expectations of access to information as well as the overall sense of cyber^5^ (in)security.^6^ The proper alignment of competing interests and practices is the premise of a functioning establishment of human rights and freedoms online. In case of failure to appropriately consider the system of data exchange, personal data protection guarantees remain assurances in letter.

Given the fundamental complexity of contemporary data processing relationships there is acute need for a systemic and structured normative approach to data protection of which personal data protection is a part. The claims of privacy and personal data protection need to be considered in the context of prevalent trends of freedom of information and expression, as well as, competing claims of ‘security’. Developing national and international personal data protection regimes needs to acknowledge the need for broad and systemic safeguards to privacy online that go far beyond explicit personal data processing regimes.

Any evaluation of norms of personal data protection should not be isolated from factors that determine or condition their implementation. As privacy is an intrinsically subjective claim, enforcing data privacy requires personal participation. This article will conclude that ‘individuality’ is the fundamental element of data privacy regulation. The density and sophistication of modern-day data processing relationships complicate the implementation and enforcement of (especially paternalistic) data protection regimes, in particular by impairing the individual’s own involvement in data processing relationships. With privacy being a commodity upon request, personal participation is a necessary condition for reinforcing privacy online. Guaranteeing, and perhaps even enforcing, personal participation is therefore a crucial consideration in national personal data protection regulation and decision-making.

## Interests and expectations: Qui bono?

### Competing rationales

The contemporary data privacy discourse is a meeting of rationales and justifications, a competition of terminologies, a convergence of technological imperatives, a contest of political and normative agendas and structures, the impossibility of extra-territorial normativism – all attaching to the mystery of human-social behavior.

Assurances of personal data protection have emerged around various rationales. In Europe, requests for clear government justification for intrusions in private life are hard-coded in the European paradigm of data protection.^7^ Privacy is centered upon the right of individual self-determination, emphasizing one’s ‘self’ against communities. Early European guarantees of privacy targeted the relationship between the individual and the Crown, the Church, and the State.^8^ At the core of the Community’s paternalistic promise of personal data protection stand personal dignity and integrity of the individual.

In the Anglo-American conception, early demands for privacy arose in the 1880s, against the push for publicity by corporate actors. [5, 6] In the US, prominent values of the ‘right of complete immunity; the right to be let alone’ have been solitude and cover against media publicity, government scrutiny and inviolability of private property. [7] The value of information, here, is subject to decision by the one who ‘owns’ it.^9^ Having acquired administrative and national security dimensions over time, the US privacy legislation remains a highly fragmented one.^10^

In technologically ambitious countries, personal data protection regimes have recently emerged as a supporting mechanism to economic growth awaited from a vibrant ICT society. [10] Yet even in jurisdictions with no significant ICT economy or contested freedom of information^11^ personal data protection laws emerge against the backdrop of national automated data processing solutions,^12^ or as export agendas, part of capacity building.^13^ Importantly, in some cases, normative guarantees for personal data protection can be traced to clustered confidentiality concerns, often in the field of financial services, or health care.^14^

One can note from these various justifications, the potential differences, in both expectations, and guarantees to personal data protection, across jurisdictions. The above accounts allow modeling personal data protection as layers of expectations with corresponding explicit regulations and policies as well as implicit assumptions of the individual, the user.

Every ascending layer contributes predictability, and transparency to automated data processing. However, every personal data protection regime requires a dense national data privacy architecture. Each layer reflects distinct practices and expectations that condition normative measures and individual behavior. As such, the table summarizes various possible frameworks of analysis and argument (Table 1).

**Table 1:** Main personal data protection incentives

Values/goals	Signified in	
Personal dignity and informational self-determination	Integrity of the individual	[Layer 6]
Protections against public scrutiny and inviolability of private property	Commercial value of data	[Layer 5]
Trust in technology	Layman’s implicit assumption	[Layer 4]
Administrative efficiency	Government functionalism	[Layer 3]
Capacity Building	Assistance and aid	[Layer 2]
Sectorial or business incentives and requirements	Business functionalism	[Layer 1]

### Technological data processing imperatives

Contributing to the complexity are the technological imperatives related to personal data processing. Data transactions have become daily routine of general government and business functions. Cross-border data flows are critical to enabling the functionality of the global financial systems, trade, logistics and transportation, crisis coordination and national defence. Only after several decades of development of international information infrastructure, and in-built ICT-dependence, has it come to the political awareness, that ICT-dependence entails threats and risks. These are still to be fully understood at national and international level. The pace of ICT development demands constant (re) assessment and mitigation of privacy risks.

Personal data protection guarantees are conditioned in the state of information society development and the accompanying discourse of cyber security. In Europe, the Network and Information Security Directive^15^ (NIS) emphasizes the essence of reliability, and security of network and information systems to the functioning of economic and societal activities, which make up the internal market. This instrument highlights the necessary link between ‘cyber’ security and personal data protection, admitting that, providing personal data guarantees needs to be made a priority, in providing ICT security. Providing ICT security is a much broader set of issues than that of personal data protection.

For a vast majority of countries, also in Europe, ICTs are imported technologies. For technologically less developed countries, proliferation of ICTs have been a result of political campaigns, run via international organizations and entities, including ITU and the World Bank.^16^ As a result, providing security of devices and systems is not a matter of solely national capacity or competence. In this context it is easy to lose sight of technological imperatives and conditioning factors of personal data processing.

Further to the difficulty of national accountability for information security, international data exchange occurs over telecommunication technologies^17^ that are by definition cross-border. In sum, national level capacity to provide the security of information and information infrastructure, is a troublesome variable as can be seen in the international cybersecurity dialogue, notably the reports of the UN Group of Governmental Experts on international information security.^18^

Furthermore, the term ‘cyber’ is obscure as to the terms, technologies and trends involved in it. Depending on the venue and actor, ‘cyberspace’ can refer to the Internet, telecommunication, dedicated military communications network, or all the above. On the same note, cybersecurity risks, most of which are associated with data, can materialize at the vendor, network operator, service or content provider level as well at the insufficient user awareness level. The vagueness of the term contributes to the lack of visibility into the nature and causing factors of personal data protection.

Similarly, technology market trends such as the *Internet of Things (IoT),* or *the Cloud* are often difficult to assess in terms of their impact on guarantees to personal data protection. As noted by the Article 29 Working Party, processing of data in the context IoT relies on the coordinated intervention of a significant number of stakeholders, such as device manufacturers – sometimes also acting as data platforms; data aggregators or brokers; application developers; social platforms; device lenders or renters.^19^ Cloud computing brings the element of extraterritoriality and the resulting concerns of effective and legal control over data. Big Data, adds dimensions of aggregation to the point - where full anonymization becomes questionable

### Actor interests

Further confusing the structure and nature of privacy risks, let alone personal data protection guarantees, are a variety of actors with diverging motivations, interests, goals and practices. For the most part, cybersecurity and risk management discourse has focused on government acts, and/or omissions. Curiously, so has the discussion of automated data processing issues the past couple of decades.^20^ After years of contested data retention practices,^21^ adequacy of national threat assessments^22^ and cross border transfers,^23^ governments, especially since the Snowden revelations, have been put in the spotlight of the current privacy discourse.^24^

Security as a stand-alone theme is at least as complex and controversial as privacy. A confrontational, either-or, approach is likely to lead to fundamental conflicts between data protection and security organizations. It is essential to contextualize the discourse of privacy within the international cyber security dialogue – without explicit universal data protection remedies and guarantees national restrictions and grants of privacy are subject to sovereign decision-making. The boundaries of privacy in the context of national security are ambiguous.

Less controversial are clear legislative guarantees and practical measures against mental and physical threats to human beings. Contested are, instead, contextual and contingent government and business-administrative claims of security as a justifying factor for abstract privacy restrictions. Regardless of the regime, or the business model in question, such assertions often share lack of transparency as a common thread. Joseph A. Cannataci, the UN Special Rapporteur on the right of privacy notes some of the highly problematic and demanding aspects: the adequacy of oversight mechanisms; the need for, and proportionality of, such measures in a democratic society; as well as the cost-effectiveness and the overall efficacy of such measures.^25^ The reported State-on-State practices of development and use of cyber capabilities to access, monitor, capture or ex-filtrate electronically transmitted or stored data.^26^

Data is military target by definition. It is a founding block for intelligence and situational awareness. Cyber military capabilities enable to deny, or manipulate adversary’s (or potential adversary’s) decision-making, through targeting not only information infrastructure, or the message/content itself, but also a cyber-persona. The latter as defined by the U.S. joint doctrine on information operations refers to “an online identity that facilitates communication, decision-making, and the influencing of audiences in the cognitive dimension”. Cyberspace capabilities are bringing online identity to the list of military targets.^27^

The private sector as well as public-private partnerships are involved and interested in gathering, analyzing and disseminating intelligence and other data of government interest. Cannataci emphasizes in his report to the Human Rights Council, the organic growth over the past two and a half decades, of appetite for all kinds of personal information by private corporations.^28^ He concludes that personal data has become a marketable and tradable commodity, meaning that the incentive for changing the business model – simply on account of privacy concerns is rather low.^29^ Private sector manipulation of personal data has glided from a side-show to the big screen.

For the foreseeable future, obtaining data requires a two-way partnership between government and the private sector. Such symbiosis will require give and take from both sides. In an appetite for control, governments are less likely to significantly sever the private sector’s ability to collect and process personal data for optimizing their production and service processes. A few prominent examples against this trend are not demonstrative of guarantees of personal privacy, but of business interests, where they overlap with normative guarantees to personal data protection. In 2016, a face-off between the US government and Apple about secrecy of iPhone communications resulted in a legally unsatisfying solution where, absent Apple cooperation, the US law enforcement decided to seek outside technical assistance to access private communications of a terrorist suspect. [18] In 2014, the US Government had to give in to Microsoft’s position that data of a US company outside the US jurisdiction is not readily available to browse for the government.

Anyone who wishes to participate in the exchange of information and ideas in the modern world of global communications is nowadays obliged to use transnational digital communication technology.^30^ The push of ‘next billion people online’^31^ is supported by businesses and their macro-economic interests. Both industry and governments are anticipating growth of online population and connectivity.^32^ The push for further development and proliferation of ICTs is a central element of national digital strategies, industry politics and development support.^33^

Legal guarantees to personal privacy are to be measured vis-à-vis the push and demand of information as well as the expectations towards societal security and stability.^34^ Just as ICTs have become an integral part of daily life, thinking about privacy and security needs to become integrated across various disciplines, within and beyond legal realms. Privacy and security in the context of ICTs is equally a matter of education policy, consumer protection, combat against crime and national industrial policy. Developing and assessing personal data protection as a stand-alone regulatory regime cannot be enough to adequately, and, or efficiently deal with risks and threats associated with present-day data processing.

## The (regulatory) framework of privacy

Current legal guarantees to privacy have evolved over several decades of societal and technological development, yet they are far from conclusive. As Prosser notes, early judicial debates in the US courts about privacy were preoccupied with the question whether the right of privacy existed at all, therefore giving little or no consideration to what it would amount to if it did. [22] Prosser’s observation reminds the sometimes hard-to-digest maxim, that judicial and regulatory steps always emerge against societal demand. What has been distilled in the course of history, may be outdated for the purposes of state of the art.

The ‘something over three hundred’ cases tried in the US courts by 1960 had distilled the ‘four torts’ of privacy,^35^ yet, offered little immediate insight into the forthcoming trend of automated data processing. Instead, those and many other cases and problem-specific regulations testify of a collage-like national data protection regime.^36^

In contrast, the EU framework of personal data protection has become thick and detailed, considering a variety of threat actors, responding to global data processing trends, and empowering the data subject – to the point where whole protectorates are put in her service at national and the community level.^37^

The European Union has a demonstrable track record as a normative data protection power, measured less in cases and more in pages of mandatory and recommended guidance.^38^ The main legal pillar in the EU data protection system is Personal Data Protection Directive (Directive 95/46/EC),^39^ updated for the purposes of online privacy by Directive 2002/58/EC.^40^ Recently omitted from this line-up is the controversial Data Retention Directive 2006/24/EC.^41^ Article 29 Working Party^42^ has issued over two hundred guidelines pertaining to specific data processing practices and issues.^43^ The Court of Justice of the EU has contributed its share^44^ with the Right to Be Forgotten ruling,^45^ push-backs on data retention^46^ and a revision of the EU-US Safe Harbour agreements.^47^

However, the latest trends in the European data protection landscape evidence of the difficulty of a harmonized solution. Invalidation of the Data Retention Directive in 2014 is an example of non-survival of a sectorial interest, although the already transposed Directive largely lives on in national legislation. The renewed EU-US Privacy arrangement, this time dubbed EU-US Privacy Shield^48^ is another. The Network and Information Systems Directive^49^ is an attempt to increase security in information systems, essential services and digital services. In line with the EU image as a personal data protection stronghold, the General Data Protection Regulation, to be in-force as of 2018, will seek to instate the requirements of privacy by design and by default.^50^ Fragmentation of personal data protection measures is also inevitable when data protection pockets are created for isolated transactions or services.^51^

Regardless of the kind of art chosen to resolve privacy from regulatory perspective, the diverging and competing elements make development and implementation of existing privacy regulations challenging.^52^ It is useful, therefore, to ask, what are the underlying considerations of the current privacy regulation. The principles^53^ enshrined in Article 6 of the Personal Data Protection Directive constitute a cornerstone of EU data protection law. The most foundational principle is the *requirement of justification* for intrusion into the privacy of the individual, whatever means employed. [26] Such justification requires disclosure from State authorities or informed consent in private data processing relations. This principle demands that relevant legal provisions and information is made clear, transparent and available in a timely manner. Other principles, notably fairness, clarity of purpose, adequacy and transparency of processing constitute and function as valuable guidelines for any personal data protection framework.

Also, in the European view, personal data protection measures are proscribed in a risk-neutral manner. It does not attach to any particular risk (f)actors. Article 17 of the Personal Data Protection Directive provides that the controller, regardless of whether it is a government or corporate or even individual actor, “must implement *appropriate* technical and organisational measures to protect personal data”. The controller must, where processing is carried out on his behalf, choose a processor providing *sufficient* guarantees in respect of the technical security measures and organisational measures governing the processing to be carried out”. Such security measures are to be implemented considering the specific operational constraints and modalities.

One must take a critical view towards how these principles are implemented in practice. With all the rules and principles to be thrown against the issue of privacy, there is a disturbing mismatch between the alleged and perceived adequacies of existing privacy regulations. A 2015 Eurobarometer on data protection showed that trust in digital environments remains low. The survey found vast majority of the respondents (81%) worried about having partial (50%) or no (31%) control over the information they provide online, while only 15% felt they have complete control.^54^ Confidence was highest towards medical institutions and lowest in online businesses, especially social media.^55^ While exact numbers and the methodology of achieving them are surprisingly obscure for information age, the National Cyber Security Alliance Consumer Privacy Index^56^ reports 92% of US consumers worried about their privacy online. Of the Australians online, 85% believe data breach notification should be mandatory for business. [27].

It is only natural that against all these concerns and risks, perceived or real, the quest for international data privacy guarantees has emerged. There is currently no universal legal instrument on personal data protection. Universally accepted guarantees to data privacy follow from numerous Human Rights instruments, notably Article 17 of the International Covenant on Civil and Political Rights.^57^ Mirror provisions can be found in regional instruments.^58^ It has been affirmed in several instances that international human rights guarantees apply online.^59^ The same can be resolved for freedom of expression,^60^ privacy,^61^ as well as freedom of opinion.^62^ However, based on different belief-systems, societal values and administrative traditions understanding and practices to content and scope of entitlements vary considerably from State to State.^63^ Recent trends in data privacy regulation outside Europe do not indicate prevalent subscription to the European standard of personal data protection.

In the context of intensifying surveillance and espionage practices, calls have been made to address guarantees to privacy at international level. In December 2013, the General Assembly adopted resolution 68/167 on the right to privacy in the digital age, initiated by Brazil and Germany.^64^ In that resolution, the Assembly affirmed that the right to privacy must be protected online, and called upon all States to review their procedures, practices and legislation related to communications surveillance, interception and collection of personal data, emphasizing the need for States to ensure the full and effective implementation of their obligations under international human rights law.

Normative-declaratory guarantees to human rights online as they are guaranteed offline are more than a blanket extension of existing guarantees – they are a call for an inventory of the catalogue of relevant concerns and an invite to discuss how existing legal instruments can be applied to adequately address online activities/presence. Human rights online will generally remain a reflection of human rights protections offline.^65^ Today, in most jurisdictions the level of privacy guarantees in relation to one’s online activities is likely worse due to the lack of full comprehension of the issue, and the lack of adequate technical and organizational capacity to protect data. Resting on the conclusion that applicability human rights online will resolve issues of data privacy is as deceiving as concluding that because we have International Humanitarian Law all human suffering has ended during conflicts.

## The need for a structured normative approach

The discussion of issues and solutions reveals the necessary building blocks and elements of a coherent personal data protection regime, while also detailing some of the difficulties of personal data control as well as individual enforcement of online privacy rights. Broadening the perspective of personal data protection to data security is most essential when seeking for a holistic normative framework for securing rights online.

A solid international framework for personal data protection is well beyond political reality. Given the fragmentation and different interpretations^66^ of the existing human rights instruments and the still considerable divide between national priorities and capabilities a universal personal data protection instrument is highly unlikely.

Developing coherent personal data protection regimes on top of international human rights instruments is paramount for giving the individual an effective remedy against malicious or negligent processing of data. At national level, regulatory protections to privacy comprise legal and non-legal frameworks beyond that of dedicated personal data protection laws. Guarantees of personal data protection materialize in full via different, yet interlinked, instruments and norms.

To take into account information society goals and objectives – personal data protection needs to be addressed with the view to online presence of the government, overall Internet penetration, services and transactions online and overall societal attitudes to the freedom of information. Personal data protection guarantees materialize in conjunction with intellectual property protections in case of databases and websites. They attach to information system security standards and general levels of awareness of basic cyber security risks and threats. A national personal data protection regime must go hand in hand with incentives and disincentives to particular online activities, for instance by criminalizing acts and omissions that result in breaches of privacy and confidentiality; or providing remedies against excessive online profiling and targeting by corporate actors. Furthermore, personal data protection guarantees need to be resolved against societal expectations of and national commitments to effective law enforcement. Finally, personal data protection regimes need to consider and intelligence the requirements of national security and defence. Military doctrines and national capabilities targeted at data necessitate personal data protection as part of national resilience plans, critical systems and services as well as national defence and military capabilities.

Such a framework is hard to achieve in one normative instrument. It is therefore essential that personal data protection is acknowledged and treated as a cross-cutting theme in national policy-making and legislation. The basic principles of privacy and personal data protection need to be upheld and adjusted in respective *lex specialis*. Accordingly, oversight and implementation of personal data protection guarantees needs to be treated as a shared responsibility, to avoid framing and resolving the issue as one of confrontation. A solid national normative approach to personal data protection and privacy in the information age is a cross-sectorial effort with due consideration of underlying values and expectations.

These observations necessitate a (re) consideration of privacy guarantees in the national legal and policy frameworks. Personal data protection has become a dimension rather than a theme. As the following chapter will show, the issue is not entirely normative. It is one of implementation. To effectively close the gap between the expectation of privacy and national reality, national strategies policies and regulations must reconcile the regulatory mantle with the core.

## Individual participation as a prerequisite of implementation

At the core of legal guarantees to privacy stands the individual. Each of us is entitled to individualized exercise of self-determination, personal identity, tolerance of unique publicity and solitude demands. Loss of individualism inevitably leads to demise of privacy. Individual interest and involvement in personal data protection process is key to preventing the expiration of privacy. What individuals do not demand, corporations and governments will/shall have no appetite to supply. From legally granted solitude, privacy is easily relegated to a chance of not being noticed in the masses of people and of data.

Several trends merit attention when considering how to maximize personal participation in data processing. The popularity of online applications and services, testifies of fusion of access to unlimited information, freedom of expression and the expectancy of privacy. Services consume data from multiple sources, obfuscating lines between the controller, purpose and benefit of such processing. As a result, it becomes difficult to grasp, let alone adequately consent to, data collecting practices online.^67^

The advent of social media has offered new ways of expressing individualism, at the cost of publicity. Some commentators compare Facebook, Twitter and YouTube to countries, not only because the size but the degree of sense of community.^68^ The freedom to think is linked to access to information and the freedom of expression. Individualism online becomes a practice of expressions – ‘status’, ‘likes’, ‘comments’, and ‘shares’. Individualism expressed through social media, creating a distinct social-virtual identity and environment (re) creates identity and the individual, paradoxically with an identity and a profile but without privacy.^69^ Such alter-individualism erodes privacy in that it escapes the solitude as a prerogative of the claim of privacy.^70^

Where individualism is turned or traded into publicity or made contingent upon one’s freedom of expression, there is an instrumental limit to how far normative privacy guarantees can be stretched. In contrast, where individuals have taken steps to seal their identity or actions from the public, there is little recourse for governments and businesses to demand access, absent clear legal cause of action.

In the latter context, a troublesome trend is ostrasizing of individuals, treating people as impersonal mass, objects of marketing or spaces to invade. Group labels such as ‘criminals’, ‘terrorists’, ‘combatants’ do not permit adequate consideration of individual circumstances and entitlements that are at the heart of privacy and personal data protection guarantees. Similarly, data protection can hardly be adequately solved for ‘consumers’, ‘students’, ‘bank account owners’ or ‘users’ – in every case a group of individuals is concerned, personal data protection guarantees become disguised and hard to fully observe by members of the group.

A useful example here is the implementation of the legal protections against automated decision-making. According to Article 15 (a) of the Personal Data Protection Directive every person has the right not to be subject to a decision, which produces legal effects concerning him or significantly affects him and which is based solely on automated processing of data. In layman practice, getting beyond ‘computer assessment’ of one’s creditworthiness, customer priority is becoming increasingly difficult. Agamben goes as far as to predict a loss of fundamental status as human, the reduction to *bare lives* that can be used or killed, as a result of further advances in artificial intelligence and automated decision-making. [33].

The time to consider these issues and developments is now. On the one hand, privacy breaches are often self-inflicted, or constitute a result of a person’s social choices. With all these trends and issues, it is important to note that the goal of all these actors and actions is hardly the assault on privacy. For governments and companies the immediate purpose is more efficient administration or a more profitable business. That privacy is taken hostage by these purposes, highlights the often-overlooked element in the granting of protections for personal data – the person herself.

At the same time, development and uses of ICTs are not a natural force. They rest on decisions. Above all, personal data protection is intended to provide an effective remedy to the data subject to defend its personal space against governments, corporate actors and other people. Inadequate consideration of privacy and data protection issues could lead to cases of state responsibility. In this context, governments need to consider due diligence standards when adopting ICTs in societal and political functions.

## Conclusion

Building personal data protection guarantees is a step-by-step process that needs to take into account the social, economic and political realities of the jurisdiction at hand. At the minimum, in jurisdictions with no strong personal data protection culture, privacy concerns can effectively be addressed in the context of confidentiality as, and when, it supports particular business models, perhaps insurance, banking, and health care in the forefront.

The interrelationship of privacy with access to information, opportunities of expression, consumerism and security is hard to break.^71^ The administrative-industrial complex^72^ is impossible to penetrate without basic privacy guarantees and understanding of the underlying technological, economic and political realities. Personal data protection regimes offer a balancing mechanism in the intertwined public-private relationships. In this regard, any jurisdiction with normative tools of personal data protection becomes a terrain of opportunity for the individual.

The test of individual protest against the polygamist concept of privacy is seminal for the future of the right to privacy. Without personal participation, data privacy becomes obsolete and cannot be effectively provided by government, no matter how liberal. Recent upgrades to personal data protection acts, especially in Europe, are expected to increase transparency about data breaches and broaden the jurisdictional surface of protest. Reinforcing the rights of individuals is one of the very few prospective remedies in the world of interconnected data and power relations. In many cases, the question becomes about the ability and willingness of individuals to pursue the already existing (or claimed to exist) rights.

However, privacy cannot be achieved through norms alone – it materializes through technological design and information architecture. Most importantly, privacy in the information age materializes through choices.

## Footnotes

1 The term ‘normative’ is used throughout the article as a synonym to ‘regulatory’, with the important emphasis on ‘regulation’ being a broader term than ‘legislation’ or ‘legally binding norms’. As many contemporary privacy issues have yet to settle in national and international consciousness and response, the word ‘normative’ is intended to underline that ‘norms’ related to privacy protection are evolving, not only as binding regulatory instruments are adopted, but, also by way of court rulings, market developments, standardization, social attitudes and non-binding normative instruments, such as policies and strategies. For a great account on norms in this respect, see Finnemore, Martha (2011) [1]

2 This article makes use of the European language of personal data protection law. The European Union is currently undergoing a personal data protection reform. This article makes reference to the regulatory framework applicable as of October 1, 2016. Occasional reference shall be made to provisions of the Regulation (EU) 2016/679 of the European Parliament and of the Council of 27 April 2016 on the protection of natural persons with regard to the processing of personal data and on the free movement of such data, and repealing Directive 95/46/EC (General Data Protection Regulation) and Directive (EU) 2016/680 of the European Parliament and of the Council of 27 April 2016 on the protection of natural persons with regard to the processing of personal data by competent authorities for the purposes of the prevention, investigation, detection or prosecution of criminal offences or the execution of criminal penalties, and on the free movement of such data, and repealing Council Framework Decision 2008/977/JHA. These instruments shall apply as of May 2018. According to Article 2(a) of Directive 95/46/EC (Article 4 (1) of the General Data Protection Regulation) personal data means any information relating to an identified or identifiable natural person (‘data subject’); an identifiable person is one who can be identified, directly or indirectly, in particular by reference to an identification number or to one or more factors specific to his physical, physiological, mental, economic, cultural or social identity.

3 According Article 2(b) of Directive 95/46/EC (Article 4 (2) of the General Data Protection Regulation) processing refers to any operation or set of operations which is performed upon personal data, whether or not by automatic means, such as collection, recording, organization, storage, adaptation or alteration, retrieval, consultation, use, disclosure by transmission, dissemination or otherwise making available, alignment or combination, blocking, erasure or destruction.

4 According to Article 15 (a) of Directive 95/46/EC Member States shall grant the right to every person not to be subject to a decision which produces legal effects concerning him or significantly affects him and which is based solely on automated processing of data intended to evaluate certain personal aspects relating to him, such as his performance at work, creditworthiness, reliability, conduct, etc.

5 See a discussion of ‘cyber’ under technological imperatives.

6 See Tikk, Eneken, Zaure Agnes (eds) (2015) [3].

7 This is underlined in the infamous Census ruling of the German *Bundesverfassungsgericht* from 1983 (BVerfG 15.12.1983. In BVerfGE 65, 1 et seq., EuGRZ 1983, 577: Urteil des Ersten Senats vom 15. Dezember 1983 auf die mündliche Verhandlung vom 18. und 19. Oktober 1983–1 BvR 209, 269, 362, 420, 440, 484/83) and explained thoroughly in the case law of the European Court of Justice and the European Court of Human Rights.

8 For a thorough account, see Kloepfer, Michael (2002) [4].

9 See Branscomb, Anne Wells (1994) [8].

10 See, e.g. Hunton & Williams, Jay, Rosemary P. (ed.) (2012) [9].

11 See “Freedom of the Net 2015 Report” [11].

12 For example Russia, Federal Law No. 152-FZ on Personal Data 2006 (Personal Data Protection Act), available at https://pd.rkn.gov.ru/authority/p146/p164/.

13 See, e.g. “Connecting_Africa: An Assessment of Progress Towards the Connect Africa Summit Goals”, African Development Bank Group (2013) [12].

14 E.g. Dubai International Financial Centre Data Protection Law, available at https://www.difc.ae/files/7814/5517/4119/Data_Protection_Law_DIFC_Law_No._1_of_2007.pdf; see also Rodrigues RJ, Wilson P, Schanz SJ (2001) [13].

15 Directive (EU) 2016/1148 of 6 July 2016 concerning measures for a high common level of security of network and information systems across the Union.

16 See Al Gore’s speech at the inauguration of ITU First World Telecommunication Development Conference – “Inauguration of the First World Telecommunication Development Conference (WTDC-94), Remarks prepared for delivery by Al Gore”, 1994, available at http://www.itu.int/dms_pub/itu-s/oth/02/01/S02010000414E05PDFE.PDF. See also the proceedings of the ITU 1998 Minneapolis Plenipotentiary, especially “ITU Efforts to Build a New Global Information Infrastructure”*,* available at https://www.itu.int/newsarchive/press/PP98/PressRel-Features/Feature5.html. For an analysis of relevant modi operandi, see Weaver, Catherine (2008) [14]. Brunsson, Nils (1989) [15].

17 For the purpose of this article, telecommunication is understood as defined by the ITU: any transmission, emission or reception of signs, signals, writing, images and sounds or intelligence of any nature by wire, radio, optical or other electromagnetic systems. ITU; International Telecommunication Convention, 1959 [16].

18 The UN Group of Governmental Experts on International Information Security convened under the aegis of the UN Disarmament and Security Committee. See the Group’s reports from 2013 and 2015 (available at www.unidir.org).

19 Article 29 Data Protection Working Party; “Opinion 8/2014 on the on Recent Developments on the Internet of Things”, 16 September 2014, available at http://ec.europa.eu/justice/data-protection/article-29/documentation/opinion-recommendation/files/2014/wp223_en.pdf, page 4.

20 OECD; Recommendation of the Council concerning Guidelines Governing the Protection of Privacy and Transborder Flows of Personal Data, C(80)58/FINAL, 23 September 1980. CoE; Convention for the Protection of Individuals with regard to Automatic Processing of Personal Data, 28 January 1981. Directive 95/46 and the surrounding public debates mainly emphasized the risk of personal data resulting from government processing.

21 See, e.g. ECtHR; S. and Marper v. The United Kingdom, 4 December 2008.; CJEU Judgment in Joined Cases C-293/12 and C-594/12.

22 See, e.g. ECtHR; Weber and Saravia v. Germany, 9 June 2006, § 78; Kennedy v. the United Kingdom, 18 May 2010; Association for European Integration and Human Rights and Ekimdzhiev v. Bulgaria, 28 June 2007; Liberty and Others v. the United Kingdom, 1 July 2008.

23 According to the EU data protection regulation, **personal data can only be transferred to countries outside the EU and the EEA** when an adequate level of protection is guaranteed. The Commission has so far concluded that outside the EU and EEA, only Andorra, Argentina, Canada, Switzerland, Faeroe Islands, Guernsey, Israel, Isle of Man, Jersey and New Zealand meet the adequacy standards (see EC; “Commission decisions on the adequacy of the protection of personal data in third countries”, available at http://ec.europa.eu/justice/data-protection/international-transfers/adequacy/index_en.htm). A special regime for exchange of personal data with the US has been in place (and contested). After, the Court of Justice of the European Union had declared the Commission’s 2000 Decision on EU-US Safe Harbour invalid on 6 October 2015 (CJEU, Judgment in Case C-362/14), the Commission adopted on 12 July 2016 by its decision a new set of exemption rules, The EU-U.S. Privacy Shield (see EC; “The EU-U.S. Privacy Shield”, available at http://ec.europa.eu/justice/data-protection/international-transfers/eu-us-privacy-shield/index_en.htm). See also Weiss, Martin A., Archick, Kristin (2016) [17].

24 On June 5, 2013 the British Newspaper the Guardian published the first article in a series based on information stolen and leaked by Edward Snowden, a former NSA contractor. See Greenwald, Glenn; “NSA collecting phone records of millions of Verizon customers daily”, The Guardian, 6 June 2013, available at https://www.theguardian.com/world/2013/jun/06/nsa-phone-records-verizon-court-order. For the timeline and themes of Snowden revelations see Aljazeera America; “Timeline of Edward Snowden’s revelations”, available at http://america.aljazeera.com/articles/multimedia/timeline-edward-snowden-revelations.html.

25 UN Human Rights Council, A/HRC/31/64, Report of the Special Rapporteur on the right to privacy, Joseph A. Cannataci, 8 March 2016.

26 See, e.g. MI5; “Director General Speaks on Terrorism, Technology and Oversight, Address by the Director General of the Security Service, Andrew Parker, to the Royal United Services Institute (RUSI) at Thames House, 8 January 2015”, available at https://www.mi5.gov.uk/news/director-general-speaks-on-terrorism-technology-and-oversight. The range and severity of threats the UK has faced over the years has meant that we have needed to build substantial security and intelligence capabilities. MI5, with our close partners in GCHQ, SIS, and the police together embody an intelligence and security effort of a quality that is the envy of many partner nations. (para 34). Also see White House; “Remarks by the President on Review of Signals Intelligence”, 17 January 2014, available at https://www.whitehouse.gov/the-press-office/2014/01/17/remarks-president-review-signals-intelligence. Today, new capabilities allow intelligence agencies to track who a terrorist is in contact with, and follow the trail of his travel or his funding. New laws allow information to be collected and shared more quickly and effectively between federal agencies, and state and local law enforcement. Relationships with foreign intelligence services have expanded, and our capacity to repel cyber-attacks have been strengthened.

27 U.S. Joint Chiefs of Staff; “Information Operations, Joint Publication 3–13”, 27 November 2012, renewed 20 November 2014, p. II-9, available at http://www.dtic.mil/doctrine/new_pubs/jp3_13.pdf. See also “Joint Publication 3–12 (R) Cyberspace Operations”, 5 February 2013, available at http://www.dtic.mil/doctrine/new_pubs/jp3_12R.pdf, describing cyberspace to consist of the layers of physical network, logical network, and cyber-persona, each representing a layer where cyber operations may be conducted. Yet cyber-persona may refer to a person, group or a state actor, an actor which has a distinct identity and respective cyber-technical attributes.

28 2016 Special Rapporteur Joseph A. Cannataci on the right to privacy, page 5, para 9.

29 2016 Special Rapporteur Joseph A. Cannataci on the right to privacy, page 5, para 9.

30 UNGA, A/69/397, Promotion and protection of human rights and fundamental freedoms while countering terrorism, 23 September 2014, page 8.

31 See, for instance, Sawers, Paul (2016) [19]. Rusli, Evelyn M. (2015) [20].

32 See, e.g. ITU Backgrounders; “Connect 2020: Setting a Global Agenda for the ICT Sector”; Plenipotentiary 2014, Busan Korea, available at https://www.itu.int/en/plenipotentiary/2014/newsroom/Documents/backgrounders/pp14-backgrounder-connect-2020.pdf.

33 The World Bank; “Maximizing Mobile, 2012 Information and Communications for Development”, 2012, available at http://siteresources.worldbank.org/EXTINFORMATIONANDCOMMUNICATIONANDTECHNOLOGIES/Resources/IC4D-2012-Report.pdf. See also UK; “Digital Inclusion Strategy”, 4 December 2014, available at https://www.gov.uk/government/publications/government-digital-inclusion-strategy/government-digital-inclusion-strategy.

34 See, e.g. White House (2011) [21].

35 Ibid. 1. Intrusion upon the plaintiff’s seclusion or solitude, or into his private affairs. 2. Public disclosure of embarrassing private facts about the plaintiff. 3. Publicity which places the plaintiff in a false light in the public eye. 4. Appropriation, for the defendant’s advantage, of the plaintiff’s name or likeness.

36 A discussion of the US personal data protection law remains beyond the margins of this article. For a good overview, see “Data Protection and Privacy in 26 jurisdictions worldwide 2014”, available at https://www.hunton.com/files/Publication/1f767bed-fe08-42bf-94e0-0bd03bf8b74b/Presentation/PublicationAttachment/b167028d-1065-4899-87a9-125700da0133/United_States_GTDT_Data_Protection_and_Privacy_2014.pdf, US pages 191–198.

37 For a great overview, see Mayer-Schönberger, Viktor (1997) [23].

38 Working Party 29 has released over 200 opinions since 1997. Opinions and recommendations available at http://ec.europa.eu/justice/data-protection/article-29/documentation/opinion-recommendation/index_en.htm.

39 Directive 95/46/EC of the European Parliament and of the Council of 24 October 1995 on the protection of individuals with regard to the processing of personal data and on the free movement of such data; OJ L 281, 23.11.1995.

40 Directive concerning the Processing of Personal Data and the Protection of Privacy in the Electronic Communications Sector, OJ L 201, 31/07/2002. As amended by Directive 2009/136/EC of 25 November 2009 amending Directive 2002/22/EC on universal service and users’ rights relating to electronic communications networks and services. Directive 2002/58/EC concerning the processing of personal data and the protection of privacy in the electronic communications sector and Regulation (EC) No 2006/2004 on cooperation between national authorities responsible for the enforcement of consumer protection laws; OJ L 337/11, 18/12/2009.

41 Directive 2006/24/EC of the European Parliament and of the Council of 15 March 2006 on the retention of data generated or processed in connection with the provision of publicly available electronic communications services or of public communications networks and amending Directive 2002/58/EC. On April 8, 2014, the Court of Justice of the European Union, the highest court of the EU, declared the data retention directive invalid. See further, CJEU; Judgment in Joined Cases C-293/12 and C-594/12, Press and Information Digital Rights Ireland and Seitlinger and Others, 8 April 2014.

42 Article 29 WP set up under the Directive 95/46/EC as an independent advisory group to address acute issues of personal data protection. It is composed of representatives of the supervisory authorities designated by each EU country; representatives of the authorities established for the EU institutions and bodies; and a representative of the European Commission.

43 For a full list, see WP29; Opinions and Recommendations, available at http://ec.europa.eu/justice/data-protection/article-29/documentation/opinion-recommendation/index_en.htm

44 For a good overview of ECJ rulings related to personal data protection, see Laudati Laraine (2016) [24].

45 CJEU; Case C-131/12, Google Spain SL and Google Inc. v Agencia Española de Protección de Datos (AEPD) and Mario Costeja González, 14 May 2014.

46 CJEU; Joined Cases C-293 & C-594/12, 8 April 2014, Digital Rights Ir. Ltd. v. Minister for Comm. Marine & Natural Res., paras. 69–73.

47 CJEU; Case C-362/14, Maximillian Schrems v. Digital Rights Ireland Ltd., 13 November 2015. European Commission decision from 12 July 2016. Privacy Shield replaces the earlier ‘Safe Harbour agreement’ invalidated by ECJ in October 2015, after it found that Safe Harbor failed to meet EU data protection standards, in large part because of the U.S. surveillance programs.

48 Ibid.

49 See Directive (EU) 2016/1148 of 6 July 2016 concerning measures for a high common level of security of network and information systems across the Union.

50 See Article 25 of the Regulation (EU) 2016/679 of the European Parliament and of the Council of 27 April 2016 on the protection of natural persons with regard to the processing of personal data and on the free movement of such data, and repealing Directive 95/46/EC, OJ L 119, 4.5.2016. See also, Allen & Overy (2016) [25].

51 Dubai International Financial Centre Data Protection Law embodies international best practice standards in line with EU directives and OECD guidelines. See “DIFC Data Protection”, available at https://www.difc.ae/laws-regulations/data-protection.

52 Such as lack of review of data prior to publication and impossibility of a quality consent and grave limitations to anonymity in the context of IoT; lack of awareness about presence of data processing equipment or the identity of the data controller in case of drones; lack of effective control over data or lack of jurisdiction transparency in case of cloud computing. For a detailed discussion, see Article 29 Working Party Opinions: Opinion 8/2014 on the Recent Developments on the Internet of Things (2014), Opinion 05/2012 on Cloud Computing (2012), Opinion 01/2015 on Privacy and Data Protection Issues relating to the Utilisation of Drones (2015).

53 According to Article 6, personal data must be (a) processed fairly and lawfully; (b) collected for specified, explicit and legitimate purposes and not further processed in a way incompatible with those purposes. Further processing of data for historical, statistical or scientific purposes shall not be considered as incompatible provided that Member States provide appropriate safeguards; (c) adequate, relevant and not excessive in relation to the purposes for which they are collected and/or further processed; (d) accurate and, where necessary, kept up to date; every reasonable step must be taken to ensure that data which are inaccurate or incomplete, having regard to the purposes for which they were collected or for which they are further processed, are erased or rectified; (e) kept in a form which permits identification of data subjects for no longer than is necessary for the purposes for which the data were collected or for which they are further processed. Member States shall lay down appropriate safeguards for personal data stored for longer periods for historical, statistical or scientific use.

54 EC; “Data protection Eurobarometer out today”, 24 June 2015, available at http://ec.europa.eu/justice/newsroom/data-protection/news/240615_en.htm.

55 Ibid.

56 StaySafeOnline; “Truste/National Cyber Security Alliance U.S. Consumer Privacy Index 2016 Infographic”, available at https://staysafeonline.org/stay-safe-online/resources/truste-national-cyber-security-alliance-us-consumer-privacy-index-2016-infographic.

57 UN; International Covenant on Civil and Political Rights, adopted 1966, in force 1976, Article 17. UNGA, Resolution 217 A, The Universal Declaration of Human Rights, 1948. It provides that “no one shall be subjected to arbitrary or unlawful interference with his or her privacy, family, home and correspondence, nor to unlawful attacks on his or her honour and reputation”. It further provides that “everyone has the right to the protection of the law against such interference or attacks”.

58 UDHR, Art. 19. *See also* ICCPR, Art. 19(2); ECHR, Art. 10(1); ACHR, Art 13(1).

59
*See,* e.g., Human Rights Council resolution 26/13, 20 June 2014; UNGA, A/RES/68/167, 21 January 2014, para. 10; Council of Europe Convention on Cybercrime, preamble, Art. 15.1; Deauville Declaration of the G8 Countries, Art. 10, 26–27 May 2011; Agreement between the Governments of the Member States of Shanghai Cooperation Organization on Cooperation in the field of International Information Security, Art. 4(1), 16 June 2009; International code of conduct for information security, A/69/723, 13 January 2015, Art. 2(1).

60 UDHR Art. 19; ICCPR Art. 19(2); ECHR Art. 10; ACHR, Art. 13; ACHPR Art. 9. *See also* Human Rights Committee, General Comment No. 34, para. 12 (Nov. 2, 1999); Report on the right to freedom of opinion and expression, 2011, paras. 20–22; Report on the right to freedom of opinion and expression, 2015, para. 11; EU Human Rights Guidelines on Freedom of Expression Online and Offline, para. 16, 18, May 12, 2014.

61
*See* UDHR Art. 12; ICCPR Art. 17; CRC, Art. 16; CRPD, Art. 22; Convention on the Protection of the Rights of All Migrant Workers and Members of Their Families, Art. 14. *See also* ECHR Art. 8; ACHR Art. 11. Convention for the Protection of Individuals with regard to Automatic Processing of Personal Data, CETS No. 108, 1 October 1985; UNHRC, A/HRC/23/40, Report of the Special Rapporteur on the promotion and protection of the right to freedom of opinion and expression, Frank La Rue, 17 April 2013, paras. 11, 79; UNHRC; A/HRC/27/37, The right to privacy in the digital age – Report of the Office of the United Nations High Commissioner for Human Rights, 30 June 2014, para. 14. Council of Europe, Declaration on Freedom of Communication on the Internet, Principle 7 (2003); *R v. Spencer*, 2014 SCC 43 para. 62 (2014); *Totalise Plc v. The Motley Fool Ltd. & Anor* EWHC 706 (QB) (19 February 2001); *Sheffield Wednesday Football Club Ltd. and others v. Hargreaves* EWHC 2375 (QB); *Oberlandesgericht Hamm*, Case No. I-3 U 196/10 (3 October 2011).

62 UDHR, Art. 1; ICCPR, Art. 19(1).

63 Various assessments and tools are out there to assess and examine national practice and performance in development and use of ICTs. See, for instance The Cyber Readiness Index (available at http://www.potomacinstitute.org/images/CRIndex2.0.pdf), ICT Development Index (available at www.itu.int/net4/ITU-D/idi/2015/), Global Cybersecurity Index (available at www.itu.int/dms_pub/itu-d/opb/str/D-STR-SECU-2015-PDF-E.pdf), Freedom of the Net index (available at https://freedomhouse.org/report/freedom-net/freedom-net-2015), UN E-Government Survey (available at https://publicadministration.un.org/egovkb/en-us/Reports/UN-E-Government-Survey-2016), Networked Readiness Index (available at http://reports.weforum.org/global-information-technology-report-2015/network-readiness-index/), Index on Digital Life (available at http://indexdigitallife.telefonica.com) as well as regional comparative assessments (Cyber Maturity in Asia-Pacific Region, available at www.aspi.org.au/publications/cyber-maturity-in-the-asia-pacific-region-2015/Cyber-Maturity-2015.pdf and OAS Cybersecurity Report available at www.cybersecurityobservatory.com).

64 This instrument was co-sponsored by 57 Member States and adopted without a vote.

65 See, e.g., European Commission (2015) [28].

66 See, for instance the US view on the implementation of the ICCPR: States Party to the Covenant should wherever possible refrain from imposing any restrictions or limitations on the exercise of the rights recognized and protected by the Covenant, even when such restrictions and limitations are permissible under the terms of the Covenant. See, U.S. reservations, declarations, and understandings, International Covenant on Civil and Political Rights, 138 Cong. Rec. S4781–01, daily ed., April 2, 1992. In contrast, Russia has emphasized that the exercise of rights and freedom in information space is contingent of relevant national laws and regulations. See, Draft International Code of Conduct for Information Security submitted to the United Nations on September 12, 2011 by China, Russia, Tajikistan and Uzbekistan.

67 See also Working Party 29; Opinion 15/2011 on the definition of consent, 13 July 2011, available at http://ec.europa.eu/justice/data-protection/article-29/documentation/opinion-recommendation/files/2011/wp187_en.pdf.

68 See, e.g. VincosBlog; “World Map of Social Networks”, January 2016, available at http://vincos.it/world-map-of-social-networks/.

69 On the impact of networks see for example Slaughter, Anne-Marie (2004) [29]. On the possibility cosmopolitan community Bull, Hedley (1977) [30]. On virtual identities Rosen, Christine (2007) [31]. as well as Holt, Douglas (2016) [32].

70 See Brown, Aaron, “Police say YOU should avoid THIS Facebook feature”, Express, 13 May 2016, available at http://www.express.co.uk/life-style/science-technology/670120/Facebook-Police-Emoji-Reactions-Like-Button. Griffin, Andrew; “Facebook Reactions: Belgian police warn citizens not to react to posts on social media”, Independent, 13 May 2016, available at http://www.independent.co.uk/life-style/gadgets-and-tech/news/facebook-reactions-belgian-police-warn-citizens-not-to-react-to-posts-on-social-media-a7027786.html .

71 For a discussion how privacy, freedom of information and expression, national security and International stability interact, see See Tikk, Eneken, Zaure Agnes (eds) [3].

72 Paraphrasing the notion of military and industrial complex President Eisenhower coined in his farewell address.
